# Effect of a Novel Adaptive Handle Design on the Ergonomic Performance of Periodontal Curettes in Dental Hygienists with and without Musculoskeletal Disorders: A Pilot Clinical Study

**DOI:** 10.3390/dj12080253

**Published:** 2024-08-13

**Authors:** Cherie Wink, Susan Meishan Yang, Ali A. Habib, Kairong Lin, Thair Takesh, Petra Wilder-Smith

**Affiliations:** 1 Beckman Laser Institute, University of California Irvine, Irvine, CA 92612, USA; cwink@hs.uci.edu (C.W.); susanmy@uci.edu (S.M.Y.); kaironl@uci.edu (K.L.); ttakesh@uci.edu (T.T.); 2 Department of Neurology, University of California Irvine, Orange, CA 92868, USA; aahabib@hs.uci.edu

**Keywords:** adaptive curette, dental hygiene, musculoskeletal disorder, ergonomics, silicone handle, resin handle, electromyography

## Abstract

(1) Background: Musculoskeletal disorders (MSDs), discomfort, fatigue, pain, and other acute and chronic work-related injuries are common among dental clinicians. Hand instruments constitute a primary risk factor for these conditions. The overall goal of this study was to compare in dental hygienists with healthy hands, and in those with MSDs, the effect of three different handle designs on instrumentation-related muscle work, comfort, fatigue, and quality of tactile feedback. (2) Methods: Clinicians tested three periodontal curettes: one with a novel adaptive silicone handle, another with a rigid resin handle, and the third with a rigid silicone handle. Ten hygienists—five with MSDs and five without—each scaled three typodonts using the three different curettes. Statistical analysis was performed using a General Linear Model (GLIM) and Tukey’s post hoc test, and a significance level of *p* < 0.05 was implemented. (3) Results: On average, mean comfort and fatigue across all instruments were significantly worse in testers with MSDs, who also expended significantly more work to complete the same task. In all testers, a novel adaptive handle design was associated with significantly reduced total muscle work and post-instrumentation fatigue, as well as better comfort than conventional rigid handle designs. (4) Conclusions: An adaptive curette handle design demonstrated significantly better ergonomic outcomes than conventional rigid curette handle designs. Hygienists with MSDs expend significantly more muscle work during dental instrumentation.

## 1. Introduction

Musculoskeletal disorders (MSDs) and other repetitive stress injuries (RSIs) are common in hygienists, especially in those who work full time or who have practiced clinically over many years [1,2,3,4,5,6,7,8,9,10,11,12]. Most hygienists report symptoms consistent with musculoskeletal overloading or long-term chronic injury [13]. Thus, it comes as no surprise that many hygienists can only work part-time due to pain and disability from work-related MSDs, which adversely affect their ability to instrument effectively [11,14,15].

The majority of dentists also report musculoskeletal pain and dysfunction [13,14,15,16,17,18,19,20,21,22,23,24,25,26,27,28,29,30,31], and many eventually reduce working hours or retire because of work-related musculoskeletal disorders [15]. This situation affects the personal and professional aspects of dental clinicians’ lives. One study estimated that up to 48.1% of dental clinicians experience MSD-related insomnia and stress-related conditions. Moreover, 6.4–46.5% of these individuals report a prevalence of frequent pain, and 33% report a reduced ability to work. Poor sleep, frequent, recurrent, and severe stress, and multi-site pain all adversely affect dentists and their ability to work [32]. In addition to causing disability, pain, and suffering, these conditions also have considerable detrimental effects on the financial well-being of dental clinicians, resulting in an estimated annual loss of earnings of USD131 million [33].

Throughout their training, dental clinicians are taught how to best fit their hands and fingers to rigid dental tools whose shapes are not adapted to the actual anatomy of the hands and fingers. The need to maintain non-anatomical and non-ergonomic positions over long periods of repetitive, high-force, and precise instrumentation may well be an important contributor to musculoskeletal discomfort and injury in these clinicians. In this study, the investigators evaluated the ergonomic performance of a dental hand instrument specifically designed to conform to the shape of the hand and the fingers, and to divert some of the forces of instrumentation to the larger muscles and surfaces of the hand.

The goals of this in vivo study were to (1) compare the ergonomic performance of three periodontal curettes with different handle designs and materials and to (2) compare these outcomes in hygienists with and without MSDs.

## 2. Materials and Methods

This protocol was granted exempt status after review by the University of California, Irvine’s IRB, as only de-identified data were collected and used. 

### 2.1. Testers

Ten right-handed hygienists, with a minimum of 5 years of clinical practice for at least 3 days per week, were recruited for this study. Study participants were recruited by phone calls, e-mails, and text messages. They were all experienced current or previous periodontal instrumentation instructors at local Schools of Dental Hygiene (University of Southern California, West Coast University, Cypress College, Cerritos College, San Juaquin Valley College, and Concorde College Schools of Dental Hygiene). Five of the testers had not experienced any injuries or known disorders of their fingers, hands, or wrists within 6 months of study begin. The remaining 5 testers had been diagnosed with an instrumentation-related ongoing musculoskeletal disorder (MSD), repetitive stress injury (RSI), or chronic pain/problems in their hands.

The inclusion criteria for all testers were as follows:

− Dental hygienists;
− Right-handed.

The inclusion criteria for the MSD group were as follows:

− ≥1 year of chronic intermittent or continuous symptoms of a medically diagnosed MSD, RSI, or similar, including discomfort, aching, numbness, tingling, burning, stiffness, and decreased range of motion.

The exclusion criteria for all testers were as follows:

− Any prescribed or over-the-counter (OTC) analgesics or anti-inflammatory medications.

### 2.2. Protocol

Hygienists in each of the two groups (“MSD” and “no MSD”) were randomized for sequences of instrument use by means of research randomizer software (randomizer.org, last accessed on 14 August 2023). Each clinician tested all 3 of the curette models, (Barnhart 5/6), working sequentially on 3 typodont models, and scaling 1 model with each type of curette. Because of concerns about potential fatigue overlap between study segments, a rest period between each segment was incorporated into the protocol. The duration of this rest period has been validated in a prior study [34], which found that a 20 min break allows all evaluation parameters to return to baseline. Because the adaptive handle was new to all the testers, they were shown a 20 s instructional video demonstrating its use. Then, the hygienists were given 5 min to accustom themselves to the instruments and to ask questions of the supervising hygienist. Each curette was sharpened by the same experienced dental hygiene instructor each time before it was used. 

The typodont models were prepared for this study precisely eighteen hours before use because artificial calculus increases in hardness over time. The artificial biofilm (Occlude Green Marking Spray, Pascal International, Bellevue, WA 98009, USA) and calculus (Dental Calculus Set, Kilgore International Inc., Coldwater, MI 49036, USA) were applied supra- and sub-gingivally to 32 artificial typodont teeth by the same researcher in a standardized fashion. Once the artificial calculus had hardened, the teeth were mounted in a typodont model, which was then attached to a manikin (Kilgore International Inc., Coldwater, MI 49036, USA). Each manikin was fixed onto an adjustable clinical dental chair. The hygienists were allowed to change their seating position and adjust the typodont position as needed throughout the study. Because each adjustment temporarily affected the surface electromyography (sEMG) traces that were being recorded during instrumentation, the researchers observing the clinicians noted the time of each adjustment. This allowed the resultant disruptions in the sEMG trace to be identified during data extraction and analysis.

The testers were instructed to complete periodontal instrumentation as if they were working on a live patient. They scaled each typodont for a total of 8 min, spending 1 min instrumenting each typodont segment, with the goal of removing as many of the simulated plaque and calculus deposits as possible during this time. The same scaling sequence was maintained for each of the 3 scalers: (1) lower anterior sextant facial surfaces, (2) lower anterior sextant lingual surfaces, (3) upper anterior sextant facial surfaces, (4) upper anterior sextant lingual surfaces, (5) lower right sextant buccal surfaces, (6) lower left sextant buccal surfaces, (7) upper right sextant buccal surfaces, and (8) upper right sextant lingual surfaces. 

### 2.3. Instruments

**Figure 1:**
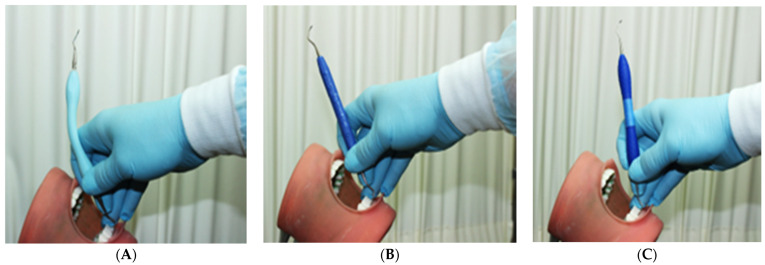
Dental curettes tested in this study: (**A**) prototype adaptive scaler; (**B**) conventional rigid resin scaler; (**C**) conventional scaler featuring a silicone-covered rigid handle.

The characteristics of the 3 universal curettes (Barnhart 5/6) with stainless steel tips that were used in this study are presented in Table 1, and representative photos of the curettes during testing are shown in Figure 1. A brief description of each instrument tested is provided below:

Curette A: a prototype universal curette (Barnhart 5/6), whose novel handle design features a flexible, universally adjustable core which allows the instrument handle to adapt to the curvature of the hand and fingers. A silicone overlay of the handle provides a cushioned, thermally insulated grip. The prototype was fabricated in our Engineering and Materials Science laboratory.

Curette B: a widely used conventional universal curette (Barnhart 5/6) with a rigid resin handle (Paradise Dental Technologies, Missoula, MT 59808, USA).

Curette C: a widely used conventional universal curette (Barnhart 5/6) featuring a cushioned, thermally insulated, silicone-covered rigid handle (Iris 4696-500 0619, Benco Dental, Pittston, PA, USA).

The testers could not be blinded regarding the different instruments that they were testing because of the characteristic appearance of each scaler. However, all data extraction and analysis were performed by a blinded researcher.

### 2.4. VAS Surveys and Open-Ended Comments

Immediately after the end of each arm of the clinical study, the clinicians were asked to complete 3 hard-copy VAS surveys assessing the following variables for each instrument on a scale of 0–10 (0—best, 10—worst):

− Fatigue;
− Comfort in
  - Wrist;
  - Fingers;
  - Palm.
− Quality of tactile feedback.

Finally, the questionnaires asked the testers to provide open-ended comments and to state whether they preferred any specific instrument and why. 

### 2.5. Surface Electromyography

**Figure 2:**
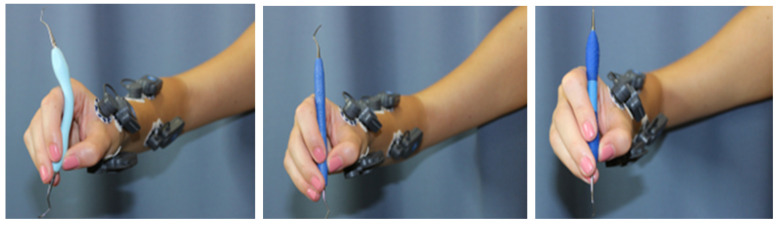
Electrode placement during testing with each instrument.

Surface EMG (sEMG) has been used over many years to assess muscle work. This technique allows researchers to chart the action potentials that are related to the activities performed by specific muscles [34,35,36,37,38]. It is especially useful for evaluating work during instrumentation, as many of the major muscles used to perform tasks such as scaling are readily accessible using surface electrodes. In this study, 4 muscles that are specifically used for gripping and manipulating dental instruments were targeted: the Abductor Pollicis Brevis (APB), the First Dorsal Interosseous (FDI), the Flexor Pollicis Longus (FPL), and the Extensor Digitorum Communis (EDC) [3,35,36,38,39,40,41]. Surface electrodes (FREEEMG, ©BTS Engineering, Quincy, MA 02169, USA) were affixed to the hand/arm above these muscles (Figure 2), and the electric action potentials generated during instrumentation were transmitted wirelessly to a Dell laptop via a USB-port dongle that connected with proprietary software on the laptop computer (FREEEMG, ©BTS Engineering, Quincy, MA 02169, USA). 

In order to quantify muscle work accurately, a reference value must be established that permits the subsequent normalization of action potential data from each sEMG electrode. For this reason, after live muscle function tests had been completed, to ensure that each electrode had been placed in the correct position on each muscle [34,42,43], the testers were asked to perform 15 s maximum voluntary isometric contractions (MVC) for each muscle separately. This trace was considered as 100% activity for that muscle and became the baseline against which all subsequent data for that muscle were normalized [44,45,46,47].

After correct electrode positioning had been confirmed and MVC data collected, sEMG signals from all 4 muscles were recorded while the testers completed the standard scaling protocol. Once the clinical testing had been completed, data processing and extraction proceeded as follows: (1) raw sEMG signals were rectified and filtered using a second-order Butterworth filter with 10 Hz high-pass cutoff frequency using BTS EMG analyzer^TM^ software (FREEEMG, ©BTS Engineering, Quincy, MA 02169, USA), and (2) total muscle activity expended by each muscle and by all muscles combined during scaling was calculated for each instrument from the integrated EMG curve [34,42,43]. One blinded investigator pre-calibrated to 95% reproducibility over a total of 50 measurements analyzed the sEMG, fatigue and comfort data.

### 2.6. Statistical Analysis

Standard SPSS 19 statistics software (IBM^®^, Armonk, NY, USA) was used to perform data analysis by means of a General Linear Model (GLIM) with pairwise tests for differences between instruments, followed by Tukey’s post hoc test. The level of significance was set at *p* < 0.05. 

## 3. Results

All testers completed this study in full compliance with the protocol. The ten female testers ranged in age from 32 to 70 years (mean age 48.1 years), whereby those without MSDs ranged in age from 32 to 55 years (mean age 41 years), and those with MSDs ranged in age from 45 to 70 years (mean age 54 years).

### 3.1. Comfort, Fatigue, and Tactile Feedback (Figure 3 and Figure 4)

#### 3.1.1. Hygienists with No MSDs (Figure 3)

Clinicians’ scores for tactile sensitivity did not differ significantly between the three test instruments. Adaptive Curette A performed significantly better than the rigid Curettes B and C in all “comfort” and “fatigue” categories. The silicone-covered Curette C performed significantly better than the resin Curette B with regard to comfort in the palm.

**Figure 3:**
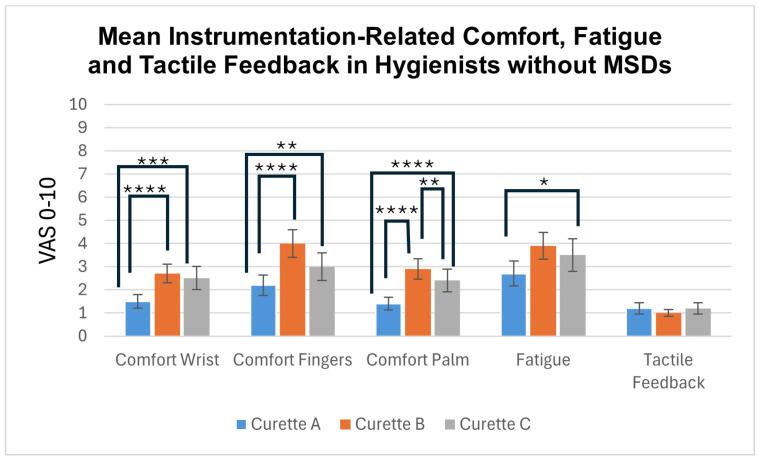
Mean instrumentation-related VAS scores (S.D.) from hygienists with no MSDs, where 0 is best and 10 is worst. Lines indicate statistically significant differences, with **** indicating *p* < 0.001; *** indicating *p* < 0.005; ** indicating *p* < 0.01; and * indicating *p* < 0.05.

**Figure 4:**
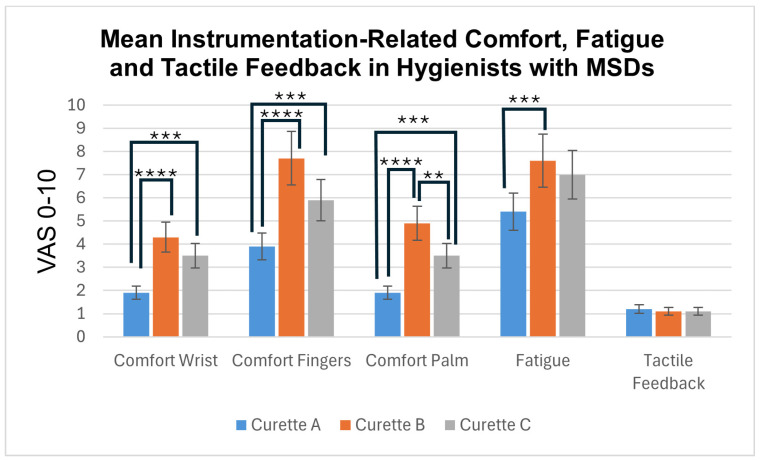
Mean instrumentation-related VAS scores (S.D.) from hygienists with MSDs, where 0 is best and 10 is worst. Lines indicate statistically significant differences, with **** indicating *p* < 0.001; *** indicating *p* < 0.005; and ** indicating *p* < 0.01.

#### 3.1.2. Hygienists with MSDs (Figure 4)

In this group of hygienists, clinicians’ scores for tactile sensitivity did not differ significantly between the three test instruments. Adaptive Curette A performed significantly better than Curette B in all “comfort and fatigue” categories in hygienists with MSDs. While these testers experienced less instrumentation-related fatigue after using the adaptive silicone Curette A vs. the rigid silicone Curette C, the difference did not reach significance. Curette C performed significantly better than the resin Curette B with regard to comfort in the palm.

#### 3.1.3. Comfort and Fatigue in Hygienists with MSDs vs. Hygienists without MSDs

On average, mean comfort across all instruments was 57–74% worse (mean 68%) in hygienists with MSDs than in those without (Table 2). 

On average, mean fatigue across all instruments was 97–100% worse (mean 99%) in hygienists with MSDs than in those without (Table 3).

### 3.2. Mean Muscle Work (Figure 5)

Mean total muscle work expended to complete the standard scaling task was significantly greater in hygienists with MSDs than in those without, independent of which curette was used. In healthy testers, significantly less muscle work was expended using the adaptive Curette A than rigid Curettes B or C. In testers with MSDs, clinicians also expended less muscle work using Curette A, and used the most muscle work using Curette B, but in these testers the differences did not reach significance. The standard deviations in this group of testers were considerable, measuring up to 28%. 

**Figure 5:**
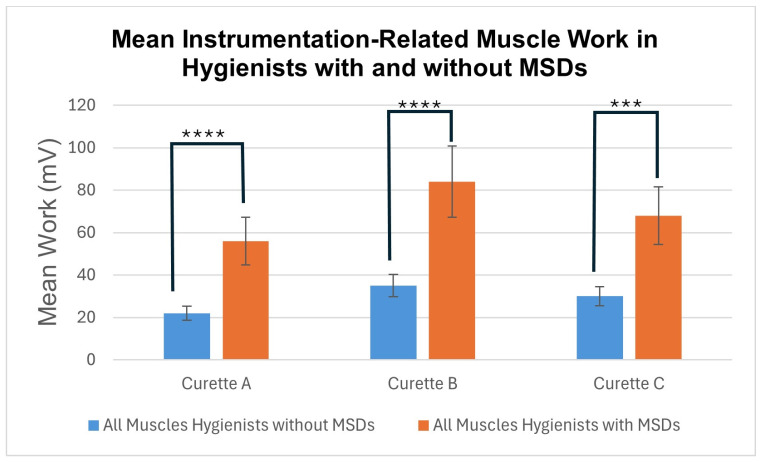
Mean instrumentation-related muscle work (S.D.) in hygienists with and without MSDs. Lines indicate statistically significant differences, with **** indicating *p* < 0.001; and *** indicating *p* < 0.005.

### 3.3. Tester Comments

Testers’ comments are shown in Table 4.

## 4. Discussion

The goal of this study was to investigate muscle work, fatigue, comfort, and tactile feedback related to a standardized scaling task by dental hygienists using three different curettes. Furthermore, this study is one of the first to investigate the role of MSDs in the ergonomic performance of dental instruments.

Previous studies investigating the ergonomic performance of dental hand instruments have evaluated variables such as specific features to spread instrumentation forces over larger areas [42,48,49,50,51,52,53], often by increasing the area of contact between the hand and the tool handle [4,48,49,50,51,52,53]. Some studies have reported on the effects of modified instrument weight, balance, shape, size, and surface properties [35,36,54,55,56,57,58]. However, the long-standing rigid handle design was always maintained in these studies. Recently, two papers for the first time reported on the ergonomic performance of an innovative bendable handle design, which allows the instrument to adapt to the shape of each clinician’s individual hand. The authors reported that this modification significantly improved all ergonomic outcomes during dental hand instrumentation [34,59]. 

The adaptive handle tested in this study incorporates into its design the known parameters that mitigate the musculoskeletal stressors inherent to dental hand instruments. Additionally, the adaptive instrument includes ergonomically favorable design features that have been incorporated into other, non-dental hand tools. For example, many studies have shown that, the greater the area of contact between a hand tool and the operator’s grasp, the more favorable will be the transfer of forces from the hand to the target surface [4,6,42,48,51,57]. Moreover, it is a well-known tenet of mechanics that spreading forces over a larger surface area reduces force/area, which will serve to reduce stress on the hands and fingers during instrument use [42,48,60]. Both of these design features are incorporated into the adaptive curette, whose bendable design allows the instrument to adapt closely to the shape of the hand and fingers, providing a larger area of contact between the hand and the instrument than can be achieved using conventional, rigid instruments. Moreover, the adaptive curette spreads instrumentation weight and forces beyond the usual areas of the hand and finger, additionally receiving support from the sides and upper surface of the index finger, as well as the back of the hand. This reduces loading per unit of area, spreading and reducing stress on the fingers and the hand during instrumentation. These features most likely contributed to the less work per time and reduced total work required to complete the set scaling task in this study, as well as the decreased fatigue and improved comfort reported associated with the use of an adaptive curette design in this study. 

A wide range of dental and non-dental studies have determined that both objective and subjective measurements are necessary for meaningful testing of the effects of instrument design on ergonomic performance [53,61,62]. Thus, ergonomic evaluations typically include neurophysiological mapping and quantification of specific muscle work, as well as validated semi-quantitative VAS markers of fatigue or comfort and unstructured user comments and assessments. The validity of this study’s mixed design is evidenced by the excellent overall agreement between the various evaluation tools that were used. The surface EMG measurements evidenced reduced work during instrumentation using the prototype adaptive curette. These findings were paralleled by the results of semi-quantitative VAS questionnaires, in which hygienists predominantly reported better comfort and less overall fatigue associated with the prototype adaptive curette vs. the rigid curettes. Moreover, rigid Curette C, which features a silicone-covered, larger-diameter handle than rigid Curette B, tended to perform better than Curette B with regard to work, comfort, and fatigue, although the differences achieved significance only with regard to comfort in the palm of the hand. These findings are in excellent agreement with the results of other studies supporting the use of wider, silicone-covered handles for dental hand instruments [4,35,37,63,64,65]. Moreover, they echo the findings of two recent studies which reported a better ergonomic performance by the prototype adaptive instrument vs. (i) a narrower-diameter, rigid, conventional stainless-steel instrument [39] and (ii) conventional curettes with rigid stainless steel, resin, or silicone handles [59].

This is one of the first published studies comparing instrumentation-related muscle work, comfort, fatigue, and tactile feedback not just between different instrument handle designs, but also in testers with and without MSDs. Because MSDs are very common in dental clinicians, and because they affect their ability to work and their quality of life, it is incumbent upon us to develop a better understanding of the causes, effects, and potential mitigations of these conditions. This knowledge should lead to better approaches for preventing and addressing work-related MSDs in dental clinicians. Both the numerical (muscle work) and semi-quantitative (comfort, fatigue, and tactile feedback) results of this study demonstrated that hygienists with MSDs perform almost double the amount of work to complete a set scaling task as their healthy colleagues. They also experience approximately twice the fatigue and half the comfort while completing the same instrumentation task These trends were seen regardless of the type of curette handle that was used, although these variables differed considerably between the various curette designs, with Cthe adaptive curette providing the most favorable ergonomic outcomes. 

In summary, a novel adaptive design for dental curettes, which integrates state-of-the-art knowledge from a wide range of disciplines, may improve the ergonomic performance of dental hand instruments. Moreover, this study determined that dental clinicians with MSDs may require considerably more muscle work and experience poorer comfort and greater fatigue than their healthy counterparts. 

The limitations of this study include the inability to blind the testers, and the use of mannikins for testing. The mannikins did, however, have the substantial advantage of providing a standardized testing substrate. Additional clinical studies with expanded investigational scope, sample size, and duration are now under way. Additional evaluation criteria have been added. They include investigations into the effect of the adaptive handle design on instrumentation efficacy and speed, as well as hand, wrist, and body positioning during instrumentation.

## 5. Practical Implication and Conclusions

(1) A novel adaptive handle design for dental hand instruments may be significantly more comfortable, require significantly less muscle work, and cause significantly less fatigue related to periodontal scaling while maintaining excellent tactile feedback. (2) Individuals with MSDs may work considerably harder and develop greater fatigue and discomfort when they perform the same clinical tasks as their healthy colleagues. (3) Extensive studies are now under way to solidify these initial findings and to develop a better understanding of novel design features to support musculoskeletal health in dental clinicians and others engaging in repetitive work with hand tools.

## Figures and Tables

**Table 1:** Overview of instruments tested in this study.

	Curette A	Curette B	Curette C
Instrument Type	Barnhart 5/6	Barnhart 5/6	Barnhart 5/6
Handle Material	Silicone-covered	Resin	Silicone-covered
Sickle Configuration	Adaptive	Rigid	Rigid
Handle Length	109 mm	107 mm	107 mm
Instrument Length	169 mm	168 mm	165 mm
Handle Diameter @ Pen Grip	11.05 mm	9.72 mm	11.59 mm
Sickle Weight	16.75 g	12.99 g	17.19 g
Blade Material	Stainless-steel	Stainless-steel	Stainless-steel

**Table 2:** Mean instrumentation-related comfort in hygienists with and without MSDs.

	Total Comfort in Hygienists with and without MSDs			
	No MSD	MSD	Diff.	%
Total (S.D.)	22.43 (3.16)	37.62 (0.42)	15.19	67.72
Curette A (S.D.)	5.05 (0.71)	7.93 (0.91)	2.88	57.03
Curette B (S.D.)	9.67 (1.03)	16.86 (1.84)	7.19	74.35
Curette C (S.D.)	7.71 (0.83)	12.89 (1.35)	5.18	67.18

**Table 3:** Mean instrumentation-related fatigue in hygienists with and without MSDs.

	Total Fatigue in Hygienists with and without MSDs			
	No MSD	MSD	Diff.	%
Total (S.D.)	10.16 (1.06)	20.17 (2.16)	10.01	98.52
Curette A (S.D.)	2.73 (0.29)	5.41 (0.69)	2.68	98.17
Curette B (S.D.)	3.92 (0.39)	7.72 (0.84)	3.8	96.94
Curette C (S.D.)	3.51 (0.38)	7.04 (0.89)	3.53	100.57

**Table 4:** Testers’ comments.

Testers without MSDs	Scaler A Adaptive Silicon Handle	Scaler B Rigid Resin Handle	Scaler C Rigid Silicon Handle
**Tester 1 (TG)**	- Like how you don’t have to grip it as hard to work * Liked the instrument	- Love how light this is - Sometimes the shank would give a little when scaling	- Comfortable grip section - Felt good * Liked the instrument
**Tester 4 (JB)**	- Feels great when it hugs my hand - Width is good. - Like the balance distribution * Liked the instrument	- I didn’t care for it. - My thumb kept slipping onto the shank which made me continuously readjust the way I held it. - Width felt fine.	- Like the shape of the instrument. - I like how it’s wider where the thumb rests against it. * Liked the instrument
**Tester 5 (KM)**	- I could really feel the difference when it was hugging my hand. It felt as if it was doing some of the work for me. - Like the thickness. * Liked the instrument	- Like the grip because it doesn’t slip. - Like the thickness. * Liked the instrument	- Like how the handle tapers into the shank. - Good size. * Liked the instrument
**Testers with MSDs**	**Scaler A** **Adaptive Silicon Handle**	**Scaler B** **Rigid Resin Handle**	**Scaler C** **Rigid Silicon Handle**
**Tester 5 (EV)** Symptoms: Numbness and tingling in ring and pinky fingers when scaling.	- Like silicon handle - Like that handle bends as it prevents thumb-flexing - Like using this straight as well * Liked the instrument best because I didn’t need to press as hard to get the job done	- Like that it’s light - Like the resin handle - Hate the step where resin meets handle because it puts more pressure on my middle finger * Liked the instrument least	- Like the texture, and silicon handle * Liked the instrument second best
**Tester 4 (LB)** Symptoms: Arthritis, trigger finger, Heberden nodes from instrumentation. Had surgery for carpal tunnel.	- I really liked this one when I adapted it to my hand. It made a huge difference for the better. - Width is good. * Liked the instrument	- Difficult to grip - My hand and fingers tired quickly	- Like the shape and weight of the instrument. - Developed discomfort and fatigue quickly using this one * Liked the instrument
**Tester 1 (ED)** **Symptoms** Arthritis, trigger finger, Hebredon nodes on joints. Had surgery for carpal tunnel and removal of nodes.	- I loved how it felt. I did not have any pain and could probably practise more with this instrument. * Liked *the instrument*	- I’m familiar with this instrument and am comfortable with it. - Sometimes the knurling feels a little aggressive. * Liked the instrument	- Like the grip section - Like the shape - Shank seems a little longer than usual * Liked the instrument
**Tester 2 (AT)** Symptoms: **Arthritis** Numbness and tingling ring and pinky fingers when scaling.	- Like silicon handle—It’s soft - Like that handle bends - Like using this straight as well * Liked the instrument best because I needed to work less	- Like that it’s light - Like the resin handle - Hate the 90-degree angle where the shank meets the handle. It hurt my fingertip. * Liked the instrument least	- Like the bulbous shape of grip portion * Liked the instrument second best
**Tester 3 (LB)** Symptoms: Thumb has pain in Policus Brevis depending on how much pinch/grip she exerts. Worsens with time spent instrumenting. General fatigue across palm whenever she instruments.	- Like handle smoothness without knurling or ridges. - Like the roundness where the handle meets the shank - Scaler feels lighter when it’s bent - Scaler doesn’t get in the way of the face - I probably wouldn’t be tired at all if I had tested this one first * Liked the instrument	- Light - Sometimes it is uncomfortable because the bumps feel pointy to my fingertips - Kind of skinny, could be thicker * Liked the instrument	- I like the hour-glass shape but felt a little too bulky. * Liked the instrument

## Data Availability

The data are available on request due to restrictions. The data presented in this study are available on request from the corresponding author. The data are not publicly available due to privacy restrictions.
